# Supplementary material for: Microwave-Assisted Synthesis, Structural Characterization and Assessment of the Antibacterial Activity of Some New Aminopyridine, Pyrrolidine, Piperidine and Morpholine Acetamides
Source: Molecules. 2021 Jan 20;26(3):533. doi: 10.3390/molecules26030533 (PMC7864198; doi:10.3390/molecules26030533)
Supplement: Supplementary file 1 [file molecules-26-00533-s001.pdf]

Microwave-Assisted Synthesis, Structural Characterization and Assessment of the Antibacterial Activity of Some New Aminopyridine, Pyrrolidine, Piperidine and Morpholine Acetamides

Abdulmajeed S. H. Alsamarrai * and Saba S. Abdulghani

Table S1. Zone diameter and minimum inhibitory concentration (MIC) for *E. coli* towards synthesized compounds **22**–**28** and referenced antibiotics.

**Bacterial species—*E. coli***									
**Synthesized Compds.**			**Synthesized Compds.**			**Synthesized Compds.**			
**Compdno.**	**Conc. (µg/mL)**	**Zone Diameter Mean Values ± SD (mm)**	**Compd no**	**Conc. (µg/mL)**	**Zone Diameter Mean Values ± SD (mm)**	**Compdno.**	**Conc. (µg/mL)**		**Zone Diameter Mean Values ± SD (mm)**
**22**	6.25	0	**25**	6.25	0	**28**	6.25		0
	12.5	* 3.4 ± 1.8		12.5	* 8.1 ± 3.5		12.5		* 6.0 ± 2.6
	37.5	* 7.7 ± 3.7		37.5	n.s 12.6 ± 4.6		37.5		* 7.5 ± 3.4
	62.5	* 10.0 ± 5.3		62.5	n.s 15.6 ± 4.8		62.5		n.s. 10.5 ± 4.5
	87.5	n.s. 13.2 ± 5.6		87.5	n.s 19.4 ± 5.0		87.5		n.s. 11.6 ± 5.0
	112.5	n.s. 14.3 ± 8.5		112.5	* 21.5 ± 5.4		112.5		n.s. 13.0 ± 6.0
	125	n.s. 16.6 ± 7.2		125	* 24.9 ± 6.0		125		n.s. 15.5 ± 7.2
**23**	6.25	0	**26**	6.25	0	Antimicrobial agents			
	12.5	7.5 ± 4.5		12.5	* 5.6 ± 2.6	Code	Disc content	Zone Diameter Mean values ± SD (mm)	
	37.5	n.s 12.0 ± 3.2		37.5	* 8.2 ± 4.1	amx	20	12.3 ± 4.0	
	62.5	n.s 16.0 ± 2.5		62.5	n.s 10.3 ± 4.3	azi	15	17.0 ± 1.4	
	87.5	* 21.7 ± 2.8		87.5	n.s 10.7 ± 2.5	doxy	30	19.0 ± 5.0	
	112.5	* 22.2 ± 3.1		112.5	n.s 12.6 ± 6.5	cep	30	13.5 ± 4.4	
	125	* 26.2 ± 4.0		125	* 10.9 ± 5.8	am	25	16.0 ± 3.0	
**24**	6.25	0	**27**	6.5		Previous studies			
	12.5	0		12.5	* 2.9 ± 1.8	Code	Disc content	Zone DiameterMean values ± SD (mm)	Ref
	37.5	* 5.8 ± 2.6		37.5	* 2.9 ± 1.9	amx	20	11–12	[36]
	62.5	* 6.5 ± 3.1		62.5	* 4.4 ± 2.2	azi	15	15–21	[37]
	87.2	* 7.2 ± 3.2		87.5	* 4.8 ± 1.9	doxy	30	11–12	[37]
	112.5	* 8.5 ± 3.8		112.5	* 5.3 ± 2.5	cep	30	±	[37]
	125	* 10.2 ± 4.2		125	* 8.2 ± 3.9	am	25	11–12	[36]

* means there are significant differences, n.s. means there are no significant differences.

Table S2. Zone diameter and minimum inhibitory concentration (MIC) for *P. mirabilis* towards synthesized compounds **22**–**28** and referenced antibiotics.

**Bacterial Species—*P. mirabilis***									
**Synthesized Compds.**			**Synthesized Compds.**			**Synthesized Compds.**			
**Compd no.**	**Conc. (µg/mL)**	**Zone Diameter Mean Values ± SD (mm)**	**Compdno**	**Conc. (µg/mL)**	**Zone Diameter Mean Values ± SD (mm)**	**Compdno.**	**Conc. (µg/mL)**		**Zone Diameter Mean Values ± SD (mm)**
**22**	6.25	0	**25**	6.25	0	**28**	6.25		0
	12.5	* 8.4 ± 1.3		12.5	* 8.0 ± 4.3		12.5		0
	37.5	n.s 14.2 ± 1.4		37.5	* 9.0 ± 5.0		37.5		0
	62.5	n.s 17.1 ± 1.1		62.5	n.s 10.2 ± 6.0		62.5		* 5.0 ± 4.0
	87.5	* 20.9 ± 2.0		87.5	n.s 11.0 ± 2.0		87.5		* 6.3 ± 1.4
	112.5	* 24.1 ± 2.9		112.5	n.s 12.0 ± 3.0		112.5		* 5.0 ± 3.3
	125	* 29.3 ± 2.2		125	n.s 13.1 ± 5.2		125		* 9.0 ± 6.0
**23**	6.25	0	**26**	6.25	0	Antimicrobial agents			
	12.5	0		12.5	0	Code	Disc content	Zone Diameter Mean values ± SD (mm)	
	37.5	* 7.0 ± 2.0		37.5	0	amx	20	12.8 ± 2.9	
	62.5	* 9.0 ± 2.5		62.5	n.s 12.0 ± 7.3	azi	15	11.3 ± 3.9	
	87.5	n.s 10.0 ± 6.3		87.5	n.s 10.0 ± 7.1	doxy	30	13.0 ± 3.2	
	112.5	n.s 11.0 ± 2.0		112.5	* 6.0 ± 7.0	cep	30	11.0 ± 3.0	
	125	n.s 12.2 ± 4.4		125	n.s 15.0 ± 13.0	am	25	7.5 ± 2.0	
**24**	6.25	0	**27**	6.5	0	Previous studies			
	12.5	* 7.4 ± 2.0		12.5	0	Code	Disc content	Zone DiameterMean values ± SD (mm)	Ref
	37.5	n.s 12.9 ± 3.0		37.5	* 6.0 ± 2.3	amx	20	11–12	[36]
	62.5	n.s 19.4 ± 7.2		62.5	* 8.2 ± 4.4	azi	15	16–21	[37]
	87.2	* 21.0 ± 7.0		87.5	* 9.0 ± 2.0	doxy	30	18–24	[37]
	112.5	* 24.0 ± 8.2		112.5	* 9.0 ± 7.0	cep	30	17–21	[36]
	125	* 27.0 ± 8.1		125	n.s 16.0 ± 2.0	am	25	11–12	[36]

Table S3. Zone diameter and minimum inhibitory concentration (MIC) for *S. pyogenes* towards synthesized compounds **22–28** and referenced antibiotics.

**Bacterial Species—*S. pyogenes***									
**Synthesized Compds.**			**Synthesized Compds.**			**Synthesized Compds.**			
**Compd no.**	**Conc. (μg/mL)**	**Zone Diameter Mean Values ± SD (mm)**	**Compdno**	**Conc. (μg/mL)**	**Zone Diameter Mean Values ± SD (mm)**	**Compdno.**	**Conc. (μg/mL)**		**Zone Diameter Mean Values ± SD (mm)**
**22**	6.25	0	**25**	6.25	0	**28**	6.25		0
	12.5	0		12.5	* 1.0 ± 1.0		12.5		0
	37.5	n.s 15.0 ± 5.0		37.5	* 3.0 ± 3.0		37.5		0
	62.5	n.s 19.0 ± 5.0		62.5	* 5.0 ± 3.2		62.5		* 8.0 ± 4.3
	87.5	* 23. 0 ± 4.2		87.5	* 6.0 ± 2.0		87.5		* 9.0 ± 2.4
	112.5	* 30.0 ± 3.9		112.5	* 7.0 ± 2.0		112.5		n.s 13.0 ± 4.0
	125	31.0 ± 4.0		125	* 8.0 ± 2.0		125		n.s 15.0 ± 2.0
**23**	6.25	0	**26**	6.25	0	Antimicrobial agents			
	12.5	0		12.5	0	Code	Disc content	Zone Diameter Mean values ± SD (mm)	
	37.5	* 4.0 ± 5.0		37.5	0	amx	20	5.8 ± 6.3	
	62.5	* 4.0 ± 3.0		62.5	* 3.0 ± 4.0	azi	15	6.5 ± 8.4	
	87.5	* 6.2 ± 2.8		87.5	* 4.0 ± 6.0	doxy	30	7.3 ± 4.2	
	112.5	* 5.1 ± 3.4		112.5	* 5.0 ± 8.4	cep	30	7.6 ± 4.3	
	125	* 7.0 ± 5.0		125	* 9.2 ± 6.0	am	25	6.4 ± 5.5	
**24**	6.25	0	**27**	6.5	0	Previous studies			
	12.5	0		12.5	0	Code	Disc content	Zone DiameterMean values ± SD (mm)	Ref
	37.5	n.s 13.0 ± 2.4		37.5	0	amx	20	30.93 ± 2.9	[44]
	62.5	n.s 15.0 ± 2.2		62.5	* 4.0 ± 2.2	azit	15	14–17	[37]
	87.2	n.s 19.0 ± 2.1		87.5	* 5.0 ± 2.0	doxy	30	13–15	[37]
	112.5	* 22.0 ± 3.0		112.5	* 7.0 ± 2.0	cep	30	±	N
	125	* 23.0 ± 3.3		125	* 8.0 ± 3.0	am	10	15–17	[37]

N: in vitro susceptibility of this antibiotic *S. pyogenes* is included in the CSLI 2017 guidelin
